# Supplementary material for: Modified FOLFIRINOX as a Second-Line Treatment for Patients with Gemcitabine-Failed Advanced Biliary Tract Cancer: A Prospective Multicenter Phase II Study
Source: Cancers (Basel). 2022 Apr 13;14(8):1950. doi: 10.3390/cancers14081950 (PMC9029419; doi:10.3390/cancers14081950)
Supplement: Supplementary file 1 [file cancers-14-01950-s001.zip › cancers-1642178-supplementary.pdf]

cancers

MDPI

Article

Modified FOLFIRINOX as a Second-Line Treatment for Patients with Gemcitabine-Failed Advanced Biliary Tract Cancer: A Prospective Multicenter Phase II Study

Yong-Pyo Lee, Sung Yong Oh, Kwang Min Kim, Se-Il Go, Jung Hoon Kim, Seok Jae Huh, Jung Hun Kang and Jun Ho Ji

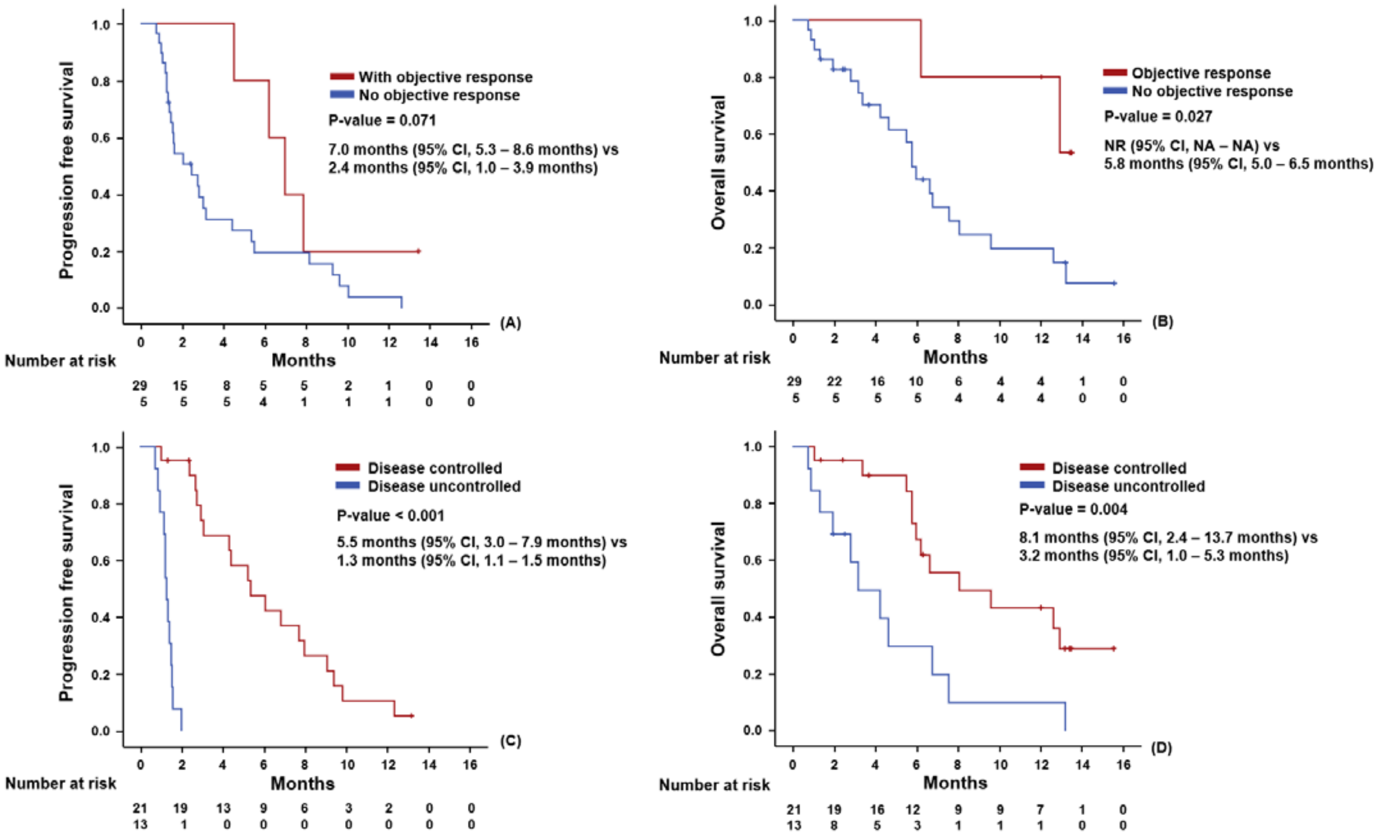

Supplementary Figure S1. Kaplan–Meier curves of progression-free survival (**A**) and overall survival (**B**) for achieving objective responses. Kaplan–Meier curves of progression-free survival (**C**), and overall survival (**D**) for obtaining disease control.

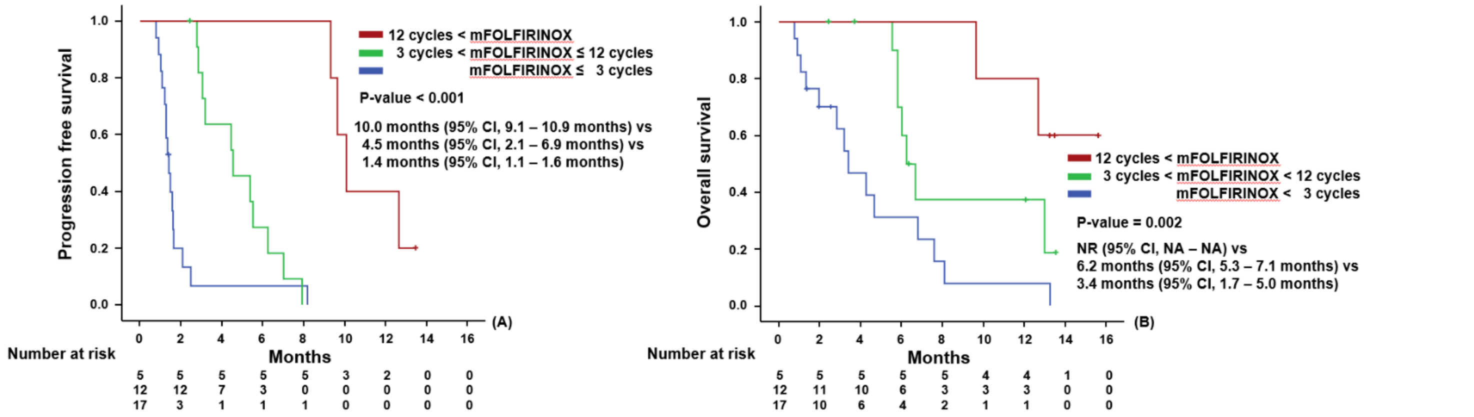

Supplementary Figure S2. Kaplan–Meier curves of progression-free survival (**A**) and overall survival (**B**) according to the number of mFOLFIRINOX administration.

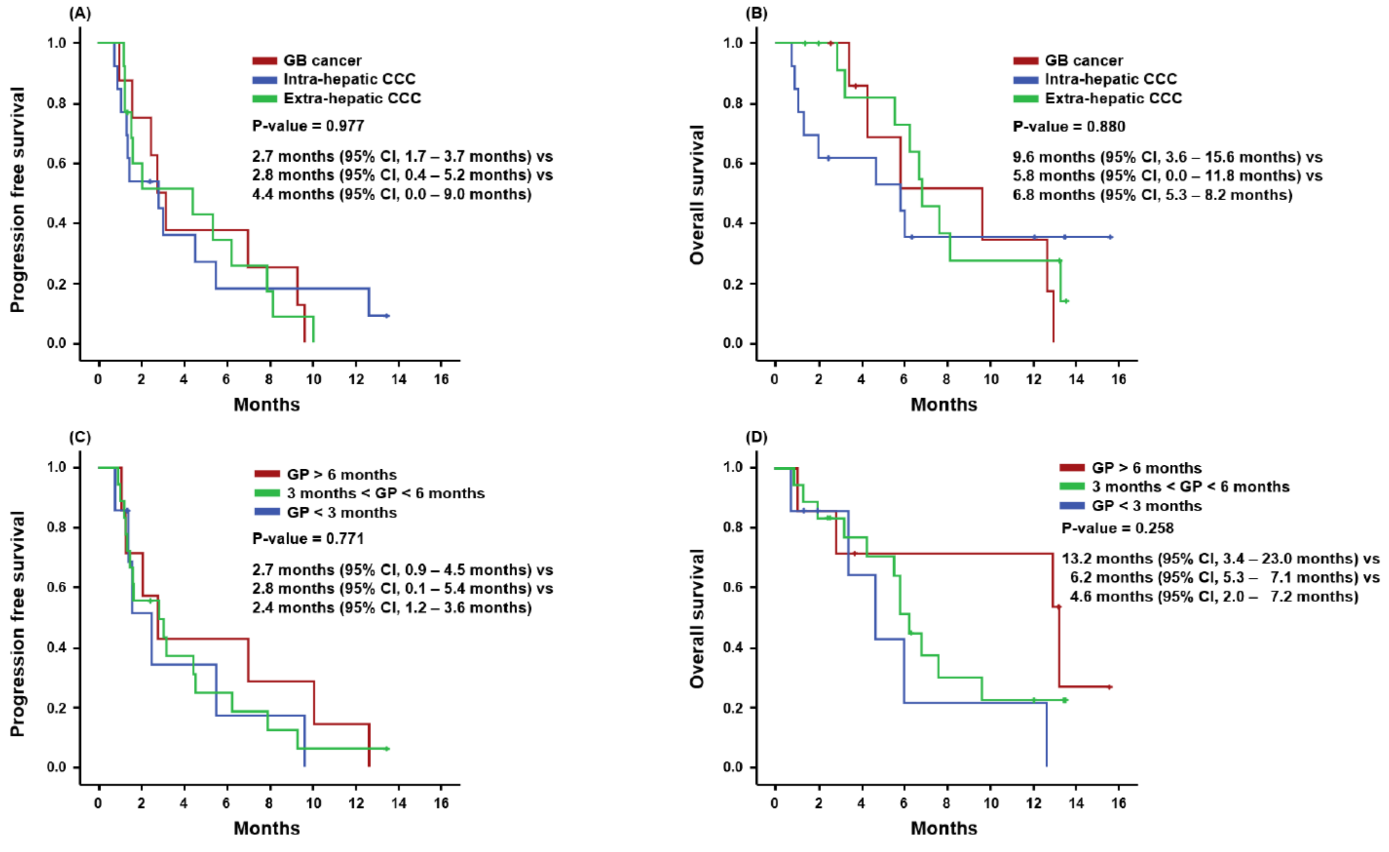

Supplementary Figure S3. Kaplan–Meier curves of progression-free survival (**A**) and overall survival (**B**) according to the type of biliary tract cancer. Kaplan–Meier curves of progression-free survival (**C**), and overall survival (**D**) according to the duration of first-line gemcitabine/cisplatin.

Supplementary Table S1. Comparison of dose, response rate, and survival results according to studies.

	Lines of treatment	Study	Disease	Chemotherapies	PFS	OS	ORR	DCR
A. Lamarca, et al.	first-line	phase III	BTC	Oxaliplatin 85 mg/m2, Leucovorin 350 mg/m2, 5-FU bolus 400 mg/m2, 5-FU infusion 2,400 mg/m2 over 46 hours	4.0 months	6.2 months	5%	33%
J. Phelip, et al.	first-line	phase II	BTC	Oxaliplatin 85 mg/m2, Irinotecan 180 mg/m2, Leucovorin 400 mg/m2, 5-FU bolus omitted 5-FU infusion 2,400 mg/m2 over 46 hours	6.2 months	11.7 months	25%	66%
A. Belkouz, et al.	second-line	phase II	BTC	Oxaliplatin 85 mg/m2, Irinotecan 180 mg/m2, Leucovorin 400 mg/m2, 5-FU bolus 400 mg/m2 5-FU infusion 2,400 mg/m2 over 46 hours	6.2 months	10.7 months	10%	66%
Liu-Fang Ye, et al.	second-line	phase II	BTC	Oxaliplatin 65 mg/m2, Irinotecan 150 mg/m2 Leucovorin 400 mg/m2, 5-FU bolus omitted 5-FU infusion 2,400 mg/m2 over 46 hours	6.7 months	13.2 months	26%	80%
Present study	second-line	phase II	BTC	Oxaliplatin 65 mg/m2, Irinotecan 135 mg/m2 Leucovorin 400 mg/m2, 5-FU bolus omitted 5-FU infusion 1,000 mg/m2/day for two days	2.8 months	6.2 months	14%	61%
Se-Il Go, et al.	second-line	phase III	PAC	Oxaliplatin 65 mg/m2, Irinotecan 135 mg/m2 Leucovorin 400 mg/m2, 5-FU bolus omitted 5-FU infusion 1,000 mg/m2/day for two days	5.2 months	9.2 months	15%	67%

BTC, biliary tract cancer; **5-FU**, 5-fluorouracil; **PFS**, progression-free survival; **OS**, overall survival; **ORR**, overall response rate; **DCR**, disease control rate; **PAC**, pancreatic adenocarcinoma.
